# Cook County Medical Society

**Published:** 1852-09

**Authors:** 


*Cook Co. Medical Society.*

TUESDAY evening, Sept. 7, Prof. Davis read a paper on erysipelas, its contagious nature, and its relation to puerperal fever.*

Dr. Brownell remarked, that in the fall of 1843 erysipelas prevailed in the region where he resided. Puerperal peritonitis was at the same time very common. One practitioner lost twelve cases in succession; he was satisfied that he communicated the disease, and retired from practice for a few weeks; on resuming his business, he had no puerperal cases, although it still prevailed in the practice of other physicians. Another physician lost a case of erysipelas,—made a *post mortem* examination,—about two weeks afterwards his own wife was confined, peritonitis supervened, terminating fatally,—the child died with erysipelas—as a general rule children escaped,—the Dr., in the mean time, had attended no cases of erysipelas after the *post mortem* till the time of his wife's confinement. Dr. B. himself was present at the *post mortem*, but took no part in the examination,—had no cases in his own practice of either erysipelas or puerperal fever.

Dr. Morfit thinks the disease non-contagious in the sense to which Prof. Davis restricts the term.

Dr. Cheeny was living at Crown Point, on Lake Champlain, in the fall of 1840, and the winter of '40-41,—the region along the lake was malarious, while a few miles back in the mountains malarious diseases were unknown. Erysipelas broke out along the lake shore, spreading rapidly and extending to the mountains; in the lower districts it was very malignant, most of the cases terminating fatally; in the mountains, although the disease appeared to be of a higher inflammatory grade, the rate of mortality was much less. All parturient women died,—physicians attending them had at the same time erysipelatous patients,—there were cases in which no medical man was in attendance, the results were equally fatal —thinks there were fewer puerperal cases in the mountains than

* See article I. of the present No.

on the lake shore,—in the two districts the disease required different treatment,—in the highlands bleeding was safe, and in many cases effectual; in the lower regions to bleed was death. All cases of wounds and injuries during this time terminated in erysipelas.

Dr. M'Arthur—In the fall of 1842 lived in Western New York, where malarious diseases had previously prevailed, but gradually diminished in frequency till about 1840, when they were unknown, —in 1842 erysipelas made its appearance, attacking the face, neck, throat, &c., and sometimes spreading over a large portion of the superior part of the body—the surface generally very sensitive, —the attacks were confined to middle aged people—in most instances terminating fatally,—puerperal fever was not common at that time.

Dr. Davis—About the same time lived in Middle and Southern New York,—saw a few mild cases of erysipelas and a *few* puerperal cases, they were not general and not malignant,—thinks the facts elicited in the discussion are interesting—it is upon facts of this nature that the doctrine of the contagiousness of the disease is based,—thinks they only prove that puerperal erysipelas is coincident with the disease in other parts of the system, and not that it is contagious,—the appearance of communication confined to periods when erysipelas prevails and all cases of injury are attacked with it,—sporadic cases do not even seem to communicate it. J.

